# Clinical and Radiological Findings in Patients with Newly Diagnosed Graves' Ophthalmopathy

**DOI:** 10.1155/2021/5513008

**Published:** 2021-05-03

**Authors:** Yakup Cevik, Hande Taylan Sekeroglu, Burce Ozgen, Kadriye Erkan Turan, Ali Sefik Sanac

**Affiliations:** ^1^Department of Ophthalmology, Nevsehir State Hospital, Nevsehir, Turkey; ^2^Department of Ophthalmology, Hacettepe University School of Medicine, Ankara, Turkey; ^3^Department of Radiology, The University of Illinois at Chicago, Chicago, IL, USA; ^4^Private Practice, Ankara, Turkey

## Abstract

**Background:**

Graves' ophthalmopathy is the most common extrathyroidal manifestation of Graves' disease. The objective of this study was to investigate the clinical ophthalmological and MRI findings in newly diagnosed Graves' ophthalmopathy.

**Methods:**

This study included 36 newly diagnosed Graves' disease patients and 23 control participants. Patients and control participants underwent detailed ophthalmologic examination. In addition, all subjects underwent orbital MRI examination; and sizes, cross-sectional areas, and signal intensities of extraocular muscles were also measured.

**Results:**

Based on MRI measurements, the mean exophthalmos in the left eye was significantly higher in the patient group when compared to those of controls (2.04 ± 0.29 vs. 1.85 ± 0.15 cm, *p* = 0.003). The mean long diameter of inferior oblique muscle in both the right and left eyes were significantly shorter in patients when compared to those of controls (*p* = 0.001, *p* = 0.002, resp.); however, the mean long diameter of superior oblique in the left eye was longer in patients than those of controls (*p* = 0.001). Patients had significantly higher superior oblique muscle signal intensity than those of controls in the right eye (*p* = 0.01). There was no significant difference for the other parameters between the patient and control groups.

**Conclusion:**

Our findings suggest that there is no obvious change in MRI examination despite clinical ophthalmological findings in patients with newly diagnosed Graves' ophthalmopathy. Unnecessary MRI examination should be avoided in this patient group due to unsatisfactory cost-effectiveness.

## 1. Introduction

Graves' disease is an autoimmune disease with a prevalence of approximately 2%, which is caused by antibodies developed mainly against thyroid stimulating hormone receptors (TSH-R) [1]. The most common extrathyroidal manifestation of Graves' disease is Graves' ophthalmopathy (GO) [2]. GO findings such as shift in the primary position, diplopia, duction limitation, optic neuropathy, and torsion may be detected in varying proportions depending on the stage of the disease, with more marked manifestations in conjunction with advanced stage [3]. The common antigens with orbital tissue and thyroid gland have been suggested to play role in the autoimmune reaction. Basically, GO is a progressive eye disease of extraocular muscles (EOM) and orbital soft tissue mediated by immune mechanisms [4].

Enlargement of EOM is an important sign of GO. The increase in muscle volume is due to muscle inflammation associated with disease activity. Several imaging modalities are used for the evaluation of EOM volume including ultrasonography, computerized tomography (CT), and magnetic resonance imaging (MRI). Ultrasonography is not reliable for the diameter measurement of too large muscles. In addition, it is limited for the imaging of the orbital apex, although it is helpful in discriminating neighboring structures [5]. Relatively few studies examined the volume measurements of muscles with computerized tomography. One of the disadvantages of CT is the exposure of the patient to ionizing radiation, which may rarely result in lens injury due to repeated imaging since GO is a chronic disease. In addition, CT provides limited information on the activity of GO [6]. It is mostly preferred for the planning of orbital decompression surgery. MRI has the disadvantages of high cost and discomfort for claustrophobic patients; however, it represents a solution for ionizing radiation and allows sections from various planes. MRI discriminates different orbital soft tissues with high resolution. It is used particularly for the cross-sectional area and volume measurements of EOM. During the last two decades, it has been used for monitoring treatment efficacy in GO. An increase in T2 relaxation time on MRI is suggested as an indication for disease activity; however, it is not a reliable indicator of the impairment of muscular function [7, 8]. Despite its several limitations, MRI is a promising modality in this setting [6].

A dramatic increase has been recently noted in the number of radiological studies involving the use of ultrasound, CT, and MRI, probably due to factors such as an aging population with multiple comorbidities as well as the general trend in related scientific studies. Today, radiological studies represent an indispensable element of both the initial assessment and follow-up of patients [9]. According to the data reported by Beinfeld and Gazelle et al. radiological imaging accounted for 19% of hospital costs between 1998 and 2000, and this figure increased by approximately 50% between 1996 and 2002 [10]. In this challenging era, evidence-based medicine has been considered a fundamental tool to assist in the decision-making process regarding proper diagnostic, medical, and interventional care strategies. However, indications for these imaging methods should be selected carefully due to the economic burden associated with their use.

There is no data that reveals the EOM alterations in newly diagnosed Graves' disease patients. This study aimed to investigate the clinical ophthalmological and MRI findings as well as evaluating EOM diameters, cross-sectional areas, and signal intensity alterations in MRI in this patient group.

## 2. Methods

### 2.1. Patients

Thirty-six newly diagnosed Graves' disease patients and 23 control participants were included in this prospective cross-sectional study. The following were the exclusion criteria for Graves' disease patients: previous radioactive iodine treatment, oral or intravenous corticosteroid therapy, orbital radiotherapy, thyroid or orbital surgery, or any known ocular disease. Control participants were selected among subjects >18 years of age who were admitted to the outpatient clinic and underwent orbital MRI examination mostly for partial sight loss or visual field cut and had no history of thyroid disease.

### 2.2. Ophthalmologic Examination

Patients and control participants underwent the following ophthalmologic examinations: best corrected visual acuity with Snellen chart, biomicroscopic anterior segment evaluation, dilated fundus examination with 90 diopter lens, intraocular pressure measurement with Goldmann applanation tonometry, light reflex, relative afferent pupil defect, Ishihara test for color vision, exophthalmos measurement with Hertel exophthalmometer (HE), Synoptophore test, binocular visual field mapping, and Hess screen test. All ophthalmological evaluations were completed by the same ophthalmologist (YC).

In addition, patients were categorized as having sight-threatening GO, moderate-to-severe GO, or mild GO, based on the European Group on Graves' Orbitopathy (EUGOGO) classification [3].

### 2.3. Magnetic Resonance Imaging

**Figure 1:**
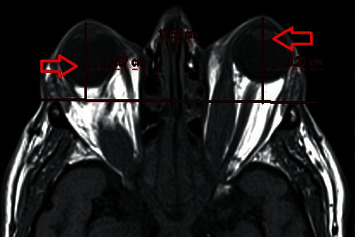
Exophthalmos measurement technique on MRI. The arrow shows the length from the foremost part of the cornea to the orbital apex.

**Figure 2:**
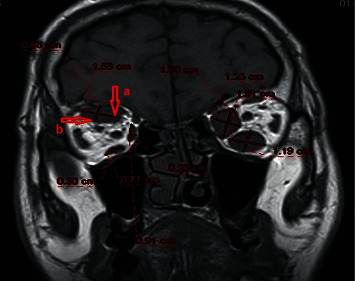
Measurement technique of muscle diameters on MRI. Arrow a shows long diameter and arrow b shows short diameter.

All subjects underwent an orbital MRI examination within seven days after the initial ophthalmological examination with a 1.5 Tesla MRI device (Tim, Siemens Medical Systems, Erlangen, Germany), using a standard head coil. The following images were obtained: transverse and coronal T1 SE (TR/TE, 500/15; 200 × 200 mm FOV; 512 × 512 matrix; 3 mm slice thickness), T2-weighted images in all three orthogonal planes (TR/TE, 3800/105; 192 × 256 matrix, 3 mm slice thickness), postcontrast transverse and coronal fat-suppressed series with similar parameters as precontrast T1 WI. Transverse scans were obtained parallel to the optic nerve and coronal sections were obtained perpendicular to the midsagittal plane. To avoid artefacts due to the contractions of EOM, patients were asked to look ahead and slightly close their eyes. Coronal sections were used for the measurements for medial rectus (MR), inferior rectus (IR), lateral rectus (LR), and superior rectus (SR) muscles, and sagittal oblique sections were used for the evaluation of superior oblique (SO) muscle and inferior oblique (IO) muscle. The signal intensity of the EOM on T2WI was recorded by manual outlining of a central region-of-interest (ROI) from the belly of the muscle. The same-size uniform ROIs were also drawn in the temporal muscle of the same side for the purposes of normalization and measurements were expressed as the ratio to that reference. MRI-based exophthalmos measurement was defined as the perpendicular distance from the interzygomatic line to the anterior surface of the eye. The measurement methods are shown in Figures 1 and 2.

### 2.4. Statistical Analyses

Statistical Package for Social Sciences (Statistical Package for Social Sciences; SPSS Inc IBM, Armonk, NY), version 21, was used for the analysis of data. For comparisons of continuous variables, Student's *t*-test for independent samples, Student's *t*-test for paired samples, Mann–Whitney *U* test, or Wilcoxon test for paired samples were used. For the comparison of categorical data, Fisher's exact test was used. The Spearman test was used to calculate the correlation coefficients and their significance. A *p* value <0.05 was considered an indication for statistical significance.

## 3. Results

In the patient group, 63.9% (23/36) were female, whereas 43.5% (10/23) of the subjects were female in the control group; however, the difference did not reach statistical significance (*p* = 0.18). The two groups did not differ regarding mean age: 40 ± 13 y (range, 19–75) for patients and 37 ± 15 y (range, 18–70) for controls, *p* > 0.05. The median (min-max) of the Clinical Activity Score (CAS) was 1 (1–3). The mean (min-max) serum TSH level was 0.9 (0.0003–3.2) mIU/L. The mean (min-max) serum-free T3 level was 5.7 (3.2–16.1) mIU/L. The mean (min-max) serum-free T4 level was 13 (4.9–25) mIU/L. There were significant correlations serum TSH level and left long diameter of IO muscle, left long diameter of SR muscle, and right long diameter of LR muscle (*r* = −0.35 *p* = 0.03; *r* = −0.37 *p* = 0.02; *r* = −0.38 *p* = 0.01). Also, serum-free T3 level and right short diameter of IR muscle was significantly correlated (*r* = 0.34 *p* = 0.03). For all other parameters evaluated, there were no significant correlations.

### 3.1. Ophthalmologic Examinations

All patients and controls had normal visual acuity, color vision, and light reflex in both eyes. In the patient group, 2 cases had relative afferent pupil defect, 2 had strabismus at PP (primary position, i.e., on straight gaze), 1 had increased intraocular pressure, 3 had limitation of eye movements, and 2 had clinical diplopia. On the other hand, none of the controls had any of these pathologies; however, the differences in frequencies did not reach statistical significance (*p* > 0.05).

In the patient group, the Hess screen test identified limitation of movement in 7, 6, 4, 4, and 4 cases in MR, IR, LR, SR, and SO muscles, respectively, for the left eye. Corresponding figures for the right eye were as follows: 5, 4, 4, 7, and 3 cases in MR, IR, LR, SR, and SO muscles, respectively. In the patient group, the Synoptophore test identified a vertical shift in 18 patients, esotropia in 17 eyes, and exotropia in 31 eyes. The Synoptophore test also identified torsion in 2 cases. On binocular visual field mapping, 11 patients had diplopia (clinical diplopia was identified in only two).

All patients were in the active phase during MRI and ophthalmologic evaluation. Based on EUGOGO classification, 2 patients had moderate to severe GO and the remaining 34 had mild GO. Two patients with moderate-severe GO had torsion and clinical diplopia. Optical neuritis was present in 2 GO patients. Baseline clinical findings of GO patients are enlightened in Table 1.

### 3.2. MRI Measurements

Based on MRI measurements, the mean exophthalmos in the left eye was significantly higher in the patient group when compared to controls (2.04 ± 0.29 vs. 1.85 ± 0.15 cm, *p* = 0.003). However, the two groups did not differ regarding the mean extent of exophthalmos in the right eye (1.99 ± 0.27 vs. 1.88 ± 0.13 cm, *p* = 0.150).

Table 2 shows the comparisons of GO patients and controls for MRI examination findings of EOM (short and long diameters, cross-sectional area, and signal intensity). Left long diameter of SO muscle was higher, and left long diameter of IO muscle and right long diameter of IO muscle were lower in patients when compared to controls (*p* = 0.001, 0.002, 0.001, resp.). In addition, patients had significantly higher SO muscle signal intensity than controls in the right eye (*p* = 0.01). The two groups did not differ with regard to other MRI findings of EOM (Table 2).

The cross-sectional area of the left IR muscle was higher in the 11 patients with diplopia on binocular visual mapping when compared to those of controls (*p* = 0.02). However, signal intensities of EOM were similar across patients with diplopia on binocular visual field mapping and controls.

In the patient group, the left and right eyes did not differ with regard to any of the EOM measurements with MRI.

### 3.3. MRI-Based versus HE-Based Measurements

In the patient group, MRI-based exophthalmos measurements were significantly higher when compared to HE-based measurements (right eye, 19.98 ± 2.70 vs. 18.38 ± 1.54 mm, *p* < 0.01; left eye, 20.42 ± 2.97 vs. 19.12 ± 1.45 mm, *p* < 0.01).

## 4. Discussion

Previous studies have investigated the MRI findings and their relations with clinical presentation in GO with inconsistent results [6, 11, 12].

In our study, the frequencies of the shift in primary position and diplopia were lower and ductions were similar in patients with mild stage GO based on EUGOGO classification compared to those of moderate to severe stage GO. Torsion was not detected on the Synoptophore examination of this patient group. All patients in the moderate-to-severe stage had torsion and diplopia. Among patients with mild stage, nine had diplopia regions not involving the center on binocular GA. Optical neuropathy has been found in 5% of patients with GO [13]. In the present study, optical neuritis was present in 6% of patients and they had moderate to severe stage based on EUGOGO classification. Meanwhile, strabismus may be observed in 17% to 51% of patients with GO, depending on the involvement of EOM [14–17]. Although all types of strabismus have been reported in GO patients, exotropia was the most common presentation in our series correlation with MRI findings.

Several studies have assessed the degree of exophthalmos in GO [18–20]. In the study by Xu et al. with 42 GO patients, the response to steroids was assessed with MRI, with exophthalmos measurements of 22.50 ± 2.17 and 20.82 ± 2.96 mm in patient groups with or without response to steroid therapy, respectively [21]. However, in contrast to ours, that study involved patients who had moderate-to-severe GO patients based on EUGOGO criteria. In our patient group, MRI measurements of exophthalmos in the right and left eyes were 19.98 ± 2.70 and 18.38 ± 1.54 mm, respectively. Asymmetric EOM involvement is a well-known phenomenon in GO [20], in line with the significant difference observed between the right and left eyes in our study.

Jankauskienė et al. reported average exophthalmos of 20.83 ± 0.64 mm with HE that was significantly higher as compared to controls (*p* < 0.001), although no information was provided on the disease stage and CAS of the study participants [22]. As compared to that study, the mean exophthalmos in the right and left eyes as measured by HE (18.38 ± 1.54 and 19.12 ± 1.45 mm, resp.) was slightly lower. In our study, the degree of exophthalmos as measured by MRI was statistically significantly higher as compared to HE measurements, for both groups.

Normally, the distance from the frontal process of the zygoma to the vertex of cornea is measured by HE [23–25]. On the other hand, by MRI, exophthalmos is measured from the perpendicular distance between the interzygomatic line and the posterior surface of the cornea (Figure 2) [26]. However, choosing an appropriate plan for HE requires skill and clinical experience, and incompatibility among clinicians can be inevitable. Lam et al. [27] reported that although the HE showed a small difference in the intraobserver, it showed a significant difference between interobservers. It can be caused by problems related to patient, observer, and device related to measurement errors of HE. However, placement of the footplates on the lateral orbital rim, the distance between the observer and the instrument, and the angle formed by the patient can contribute to this situation [28]. Furthermore, it has been reported in the literature that the measurement of HE on the two sides at the same time may not be independent of each other, and this is a factor that reduces liability to the device [29]. However, it should be borne in mind that excessive swelling and ptosis may also affect the measurement. By all these reasons explained, we think that the measurement of exophthalmos measurement by MRI can be more precise in this patient group.

The degree of EOM involvement in GO varies according to the severity and stage of the disease [30, 31]. Similar to previous reports, the MR was the most commonly affected muscle in our patient group regardless of the severity and stage of the disease. Although EOM diameter does not give an idea on disease activity and prognosis of GO [32], limited data is available on this parameter in previous studies [6, 11, 12, 22]. Similar to ours, in the study by Szucs-Farkas et al. involving 70 GO patients, the mean long diameter values for the superior muscle group, IR, MR, and LR, were 8.1, 10.0, 8.8, and 8 mm, respectively; the corresponding values for the short diameter were 4.2, 6.0, 4.2, and 3.0 mm [6]. Previous data on oblique muscle involvement is scarce. Only a case with a history of Graves' disease and IO muscle involvement was reported. The patient admitted with right blepharoptosis and on Hess screen examination limitation of movement was found at the IO region. In addition, thickening of the IO was detected on MRI examination. Following 3 days of pulse corticosteroid treatment, limitation of movement at IO region recovered and the size of IO muscle turned to normal on MRI. In that case report, it was suggested that IO involvement may be seen in GO [33]. Several studies previously showed superior oblique muscle involvement [34]. Clinical presentation of superior oblique involvement may be masked when it is together with inferior rectus involvement. Besides, the frequency of superior oblique involvement is unknown [34]. In this study, the long diameter of superior oblique muscle in the left eye was higher, long diameters of IO muscle in both right and left eyes were lower in the patient group when compared to those of controls. The fact that the left muscle long diameter and right IO muscle long diameter were lower and the cross-sectional areas in all EOM were similar in GO patients, when compared to those of the control group, may be contradictory in terms of the literature. However, our study was planned with a cross-sectional design and MRI of the patients at the time of presentation were evaluated due to GO. MRI of the same patients before the GO period is almost impossible to perform. Moreover, it is noteworthy that mostly the patients included in this study are mild stage GO. We think that this situation may be caused by the individual differences of the EOM measures in the patient and control groups. In order to verify this result, observational studies in which long-term follow-up was evaluated in this patient group are needed.

The EOM volume correlates with the cross-sectional area and has emerged as an important parameter for evaluating the degree of involvement [6, 35]. In the study by Szucs-Farkas et al., SR group and IR muscles had a higher cross-section area among GO patients as compared to controls, while there was a correlation between the cross-sectional area and the long and short diameters of all muscles [6]. On the other hand, no significant differences were observed between patients and controls in any of the muscles of any side in our study. In the studies in the literature, either the information about the stage of the disease was not provided or the patient group included in the study was composed of mostly moderate to severe GO patients [6]. Unlike other studies in our study, the patient group included in the study consisted of mostly mild stage GO patients, and therefore, no significant difference was observed in the GO patients compared to the control group.

Signal intensity is an important marker of the inflammation in the EOM. In GO patients, as a result of the increase in the inflammation in EOM, an elevation in signal intensity that correlates with CAS may occur [36]. Majos et al. showed that the signal intensity correlated with the disease severity in 40 orbitas from 20 patients, although no information was given on the disease stage [37]. In our series, the average signal intensity was significantly higher only for the right SO muscle in the patient group. Consequently, exophthalmos, cross-sectional area, and signal intensity values were lower in our study, as compared to previous reports mentioned above. This is probably due to the fact that 94% of our patients had a CAS of less than 3, corresponding to mild stage disease according to EUGOGO. Besides, we could not find clinically significant correlations between radiological parameters, both CAS and serum hormone levels, because our patients were mostly at a mild stage and had no clinically obvious presentation in terms of EOM alterations.

MRI is a very important and advanced method used to evaluate a range of morphological and functional targets. There is little doubt that MRI is one of the most powerful diagnostic tools in contemporary clinical medicine and also offers highly developed research opportunities and studies on (pathological) physiological processes. Unfortunately, it is also a costly method and increases overall healthcare costs significantly. Therefore, MRI although performed like other diagnostic tests to guide treatment, while using established clinical outcome measures is very important for evaluating its clinical use. The imaging method used in the healthcare system should respond to the request for “proof of outcome” and provide such evidence. The need to address the unsustainable increase in healthcare costs is a huge and challenging and critical issue for the general public and MRI. In our study, we tried to evaluate the effectiveness of MRI, which puts a significant cost burden on the healthcare system, in newly diagnosed GO patients. On the other hand, we concluded that there were no obvious changes in MRI and unnecessary MRI evaluation should be avoided in this patient group.

One limitation of our study relates to the fact that MRI measurements were performed manually. However, this should be considered in the light of the absence of international standards for such measurements. There are no data that reveals the MRI alterations in mild stage GO patients in the literature. This study is the first on this issue. On the other hand, a relatively small sample group was evaluated because only newly diagnosed GO patients who did not receive treatment were included in the study. The absence of significant differences between newly diagnosed mild stage GO patients and controls in terms of extraocular MRI findings leads to questions regarding the significance MRI. It appears that it may be more appropriate to avoid the unnecessary MRI studies in GO patients due to economic considerations and that a detailed ophthalmological examination may suffice for a diagnosis.

## Figures and Tables

**Table 1:** Baseline ophthalmological findings of GO patients.

Clinical findings	Number of cases (%)

Absence of color vision	0 (0%)
Absence of light reflex	0 (0%)
Torsion	2 (5.5%)
Increased intraocular pressure	1 (2.7%)
Diplopia	11 (31%)

Limitation of eye movement	Number of eyes (%)

MR	12 (17%)
IR	10 (14%)
LR	8 (11%)
SR	11 (15%)
SO	7 (10%)
IO	0 (0%)

**Table 2:** Comparisons of GO patients and controls for MRI examination findings of EOM.

	Left eye			Right eye		
	Patients (*n* = 36)	Controls (*n* = 23)	*p*	Patients (*n* = 36)	Controls (*n* = 23)	*p*
*Mean short diameter (cm)*						
Medial rectus	0.39	0.37	0.34	0.40	0.37	0.23
Inferior rectus	0.46	0.40	0.06	0.47	0.42	0.15
Lateral rectus	0.34	0.35	0.83	0.34	0.29	0.44
Superior rectus	0.36	0.34	0.35	0.35	0.33	0.67
Superior oblique	0.28	0.26	0.31	0.29	0.26	0.13
Inferior oblique	0.24	0.22	0.23	0.24	0.23	0.55

*Mean long diameter (cm)*						
Medial rectus	0.90	0.92	0.55	0.92	0.91	0.63
Inferior rectus	0.86	0.80	0.20	0.86	0.82	0.24
Lateral rectus	0.96	0.94	0.94	0.95	0.91	0.49
Superior rectus	0.86	0.86	0.59	0.84	0.86	0.59
Superior oblique	0.58	0.50	**0.001**	0.57	0.52	0.15
Inferior oblique	0.60	0.74	**0.002**	0.62	0.79	**0.001**

*Cross-sectional area (cm* ^*2*^)						
Medial rectus	0.27	0.26	0.49	0.28	0.27	0.39
Inferior rectus	0.31	0.26	0.13	0.32	0.27	0.18
Lateral rectus	0.79	0.24	0.71	0.26	0.20	0.32
Superior rectus	0.24	0.22	0.32	0.22	0.22	0.72
Superior oblique	0.12	0.10	0.32	0.13	0.10	0.11
Inferior oblique	0.12	0.13	0.30	0.12	0.14	0.12

*Signal intensity* ^*∗*^						
Medial rectus	3.49	3.56	0.95	3.52	3.54	0.36
Inferior rectus	4.03	3.50	0.16	4.08	3.43	0.10
Lateral rectus	3.68	3.83	0.73	3.61	3.65	0.73
Superior rectus	3.79	3.46	0.12	3.74	3.44	0.36
Superior oblique	3.97	3.51	0.09	4.11	3.43	**0.01**
Inferior oblique	3.45	3.19	0.42	3.28	3.15	0.83

^*∗*^Ratio to the reference signal intensity of temporal muscle at the same side.

## Data Availability

The datasets generated during and analyzed during the current study are not available due to privacy and ethical restrictions but are available from the corresponding author on reasonable request.
